# Immunologic, genetic, and ecological interplay of factors involved in allergic diseases

**DOI:** 10.3389/falgy.2023.1215616

**Published:** 2023-08-03

**Authors:** Robbi Miguel G. Falcon, Salvador Eugenio C. Caoili

**Affiliations:** Biomedical Innovations Research for Translational Health Science Laboratory, Department of Biochemistry and Molecular Biology, College of Medicine, University of the Philippines Manila, Manila, Philippines

**Keywords:** allergy, immunology, genetic, ecology, microbiome, helminths, sensitization, IgE

## Abstract

An allergic or type I hypersensitivity reaction involves a misdirected immune overreaction to innocuous environmental and dietary antigens called allergens. The genetic predisposition to allergic disease, referred to as atopy, can be expressed as a variety of manifestations—e.g., allergic rhinitis, allergic conjunctivitis, atopic dermatitis, allergic asthma, anaphylaxis. Globally, allergic diseases are one the most common types of chronic conditions. Several factors have been identified to contribute to the pathogenesis and progression of the disease, leading to distinctively variable clinical symptoms. The factors which can attenuate or exacerbate allergic reactions can range from genetic heterozygosity, the prominence of various comorbid infections, and other factors such as pollution, climate, and interactions with other organisms and organism-derived products, and the surrounding environment. As a result, the effective prevention and control of allergies remains to be one of the most prominent public health problems. Therefore, to contextualize the current knowledge about allergic reactions, this review paper attempts to synthesize different aspects of an allergic response to describe its significance in the global health scheme. Specifically, the review shall characterize the biomolecular mechanisms of the pathophysiology of the disease based on underlying disease theories and current findings on ecologic interactions and describe prevention and control strategies being utilized. An integrated perspective that considers the underlying genetic, immunologic, and ecologic aspects of the disease would enable the development of more effective and targeted diagnostic tools and therapeutic strategies for the management and control of allergic diseases.

## 1. Introduction

Adaptive immunity is a complex trait that has persisted in vertebrates across evolutionary history. It consists of humoral and cell-mediated arms, which play distinct yet interconnected roles in the body’s defense against pathogens. Between the two arms of the adaptive immunity, humoral immunity is mediated by different isotypes of immunoglobulins, each of which play a distinctive role against a wide diversity of pathogens. Of the various antibody isotypes, IgE is described as the anti-parasitic antibody isotype that participates in the clearance of helminthic parasites. To mediate this function, IgE binds to high affinity (i.e., FcεRI) and low affinity (i.e., FcεRII or CD23) receptors found on various effector cells (e.g., mast cells, basophils, eosinophils) which stimulate the release of various proinflammatory molecules involved in vasodilation, smooth muscle contraction, and other characteristic manifestations of inflammation (1). Although supposedly beneficial against parasites to prevent further damage to the host, these responses can lead to debilitating outcomes when directed against harmless environmental or dietary antigens known as allergens. Allergens are substances that range from aeroallergens found in the surrounding environment (i.e., pollen), animal detritus and dander, and certain components derived from food products (e.g., shellfish, dairy products, soybeans) which stimulate allergies.

Allergic reactions are described as immediate misdirected immune responses. Such reactions only occur across individuals who are sensitized and exposed to specific allergens. Typically, allergic sensitization or a predisposition to generate an IgE-mediated allergic response (i.e., atopy) occurs as a result of a complex interplay of different genetic factors and environmental exposures. However, studies have identified the involvement of non-IgE immune components in allergic diseases. Interestingly, allergic reactions have been characterized primarily across mammalian species despite the existence of the adaptive immune system across other vertebrates (2). This can be partially attributed to the divergence of the IgG and IgE antibody isotypes from the IgY antibody isotype characteristically found in avian and non-avian reptiles (3). In a previous study by Borges et al., IgY was shown to be involved in mediating antiparasitic immunity across avian species, homologous to the role of IgE in mammalian species (4). However, further investigation revealed that despite its structural resemblance to the IgE antibody isotype, IgY exhibits similar functional characteristics and binding kinetics to the IgG antibody isotype. Differences in the binding sites in the Fc region may be the primary basis for the differences in antibody function, leading to the divergence of the IgG (i.e., Cγ2) and IgE (i.e., Cε3) isotypes (5). Apart from IgY, the role of the IgD isotype in mucosal immunity, immunoactivation, and proinflammation has led many to postulate its potential role in allergic disease progression (6).

Many studies have focused on elucidating the interrelationship of these factors and their role in the progression and severity of allergies. However, the genetic heterogeneity of allergic individuals and the distinctive properties of their immune systems has impeded the full elucidation of the underlying aspects of allergic disease. Moreover, the elusiveness of the allergic response in terms of clinical manifestations and the molecular aspects of the disease stagnated the development of effective and widespread diagnostic and therapeutic strategies.

## 2. Current theories on allergic diseases

### 2.1. Allergic march

The development of various types of allergic diseases differs across individuals as a result of different interconnected yet highly variable intrinsic and extrinsic factors. As aforementioned, the innate heterogeneity of these etiologic factors leads to the variation exhibited across allergic individuals. Several studies attempt to characterize the progression of allergic disease development based on the factor of age. Findings suggest that certain diseases tend to predominate and occur at a greater incidence across specific age groups. This theory that describes the temporal trend of allergic disease manifestation is called the “allergic march” (7). Firstly, in the context of atopic dermatitis (AD), it was determined that there exists a causal link between onset of AD and the later manifestation of other allergic diseases. This is attributed to dysfunctionality in the physical barriers of the immune system, which serve as the primary sites of allergic sensitization and colonization of proinflammatory microbiota which are correlated with allergic disease progression. Following this is the induction of type 2 immune responses via effector Th2 cells, which renders hosts susceptible to allergic respiratory responses by upregulating airway hyperresponsiveness to aeroallergens (8). Furthermore, allergic disease progression in the context of aging, beyond the pediatric population, has also been investigated. Recent studies have demonstrated the phenomenon of inflammaging and immunosenescence among the elderly, leading to worsened disease outcomes in the context of acute and chronic inflammatory diseases (9). Moreover, studies indicate that the characteristic hallmarks of cellular aging such as oxidative stress due accumulation of reactive oxygen species, shortening and dysfunctionality of telomeres, and the increased expression of genes associated with aging have been shown to contribute to exacerbation of allergic respiratory diseases, such as allergic rhinitis and bronchial asthma (10–12). These findings suggest that targeted therapies must also consider the temporal progression of the allergy. Administering treatments outside the specific timeframe may result in suboptimal effects to alleviate disease symptoms (13).

### 2.2. Hygiene hypothesis

On the origin of allergic diseases, many theories have been proposed based on available historical evidence and evolutionary patterns. Firstly, with the advent of modern public health and sanitation practices, many disease-causing pathogens have rapidly declined in prevalence. Of significance in the context of allergic diseases are the parasitic helminths, which are known to elicit IgE-mediated immune responses. Global deworming efforts have been rampant with the aid of widespread treatment strategies and improved diagnostics being a focal agenda of various public health regimes. However, with the eventual decline of helminth populations comes the rise of misdirected immune responses, the allergic reactions. Several studies have demonstrated the immunomodulatory effect of helminth-derived extracts, indicating their possible role in the prevention of allergic diseases (14, 15). This phenomenon was first described as the “hygiene hypothesis,” by the epidemiologist, David Strachan (16, 17).

### 2.3. Old friends hypothesis

Graham Rook cites that reduced exposure to immunoregulation-inducing microbiota, which persisted across mammalian evolution, leads to poorer control over host inflammatory responses associated with various allergic diseases. He coined this proposed theory as the “Old Friends Hypothesis” to explain the underlying pathogenesis of various chronic inflammatory diseases (18–20). Rook describes the association of chronic inflammatory disorders due to a failure to sustain immunoregulation due to the absence of helminths, non-pathogenic environmental bacteria, and certain gut commensals. These microbes which he referred to as, “Old Friends” drive the expansion of specific populations of immunomodulatory regulatory T cells (Treg) and dendritic cells by the secretion and expression of specific molecules (e.g., IL-10, TGF-β, CRR4) (21–23). These immunomodulatory molecules function by directly acting on immune cells mediating allergic diseases or by downregulating signaling pathways directly involved producing a state of inflammation in the host (24).

### 2.4. Biota alteration theory

Over time, the advent of new discoveries and the growing attention on the human microbiome, the original concepts of Strachan were reshaped to consider the host ecological interactions with the endogenous microbial symbionts and the surrounding environment (19, 20). One such theory which gained popularity for integrating other inflammatory diseases and the involvement of the host microbiota is the “biota alteration theory” (25).

Based on these theories, several factors are shown to be associated with changes in the host microbiome, leading to various inflammatory diseases—including biota depleting, dirt, and other factors, which affect microbial biodiversity. Biota depleting factors include medical factors such as the use of antibiotics, (i.e., modernization of birthing practices), and clean factors which include improved sanitation and food processing technology, and modern construction. Dirt factors, on the other hand, include increased population density and developed construction practices, which increases the predominance of proinflammatory microbiota.

### 2.5. Toxin hypothesis

Beyond allergic responses to traditional airborne and dietary allergens, certain iterations of hypersensitivity reactions were historically described as allergies. A theory which illustrates the origin of toxin allergy diseases is the “toxin hypothesis of allergy” which was first described by Margie Profet (26). This theory is based on observations of enzymatic degradation of venom toxin by mast cell-derived proteases, which enhance resistance to venom allergies. The administration of phospholipase A2 (PLA2), a conserved component of bee venom, normally induces a type 2 cell-type response and group 2 innate lymphoid cell activation via the enzymatic cleavage of membrane phospholipids and release of IL-33. As a result of this IgE response to PLA2, protection against anaphylaxis from future challenge can be induced with near-lethal doses of PLA2 (27). Therefore, these findings suggest the possible role of mast cells and IgE-dependent responses in promoting innate and adaptive resistance to venom allergies, forming the basis for immunotherapeutic strategies. However, underlying factors that determine whether a bee venom-induced IgE response leads to pathologic anaphylaxis or protective immunity remains to be poorly elucidated (27–30). The investigation of possible compounds derived from other particles capable of eliciting beneficial immune responses that antagonize the pathologic manifestations of allergic disease can be exploited for the design of novel therapeutics (31, 32).

## 3. Immunologic basis of allergic diseases

### 3.1. Pathophysiology

**Figure 1:**
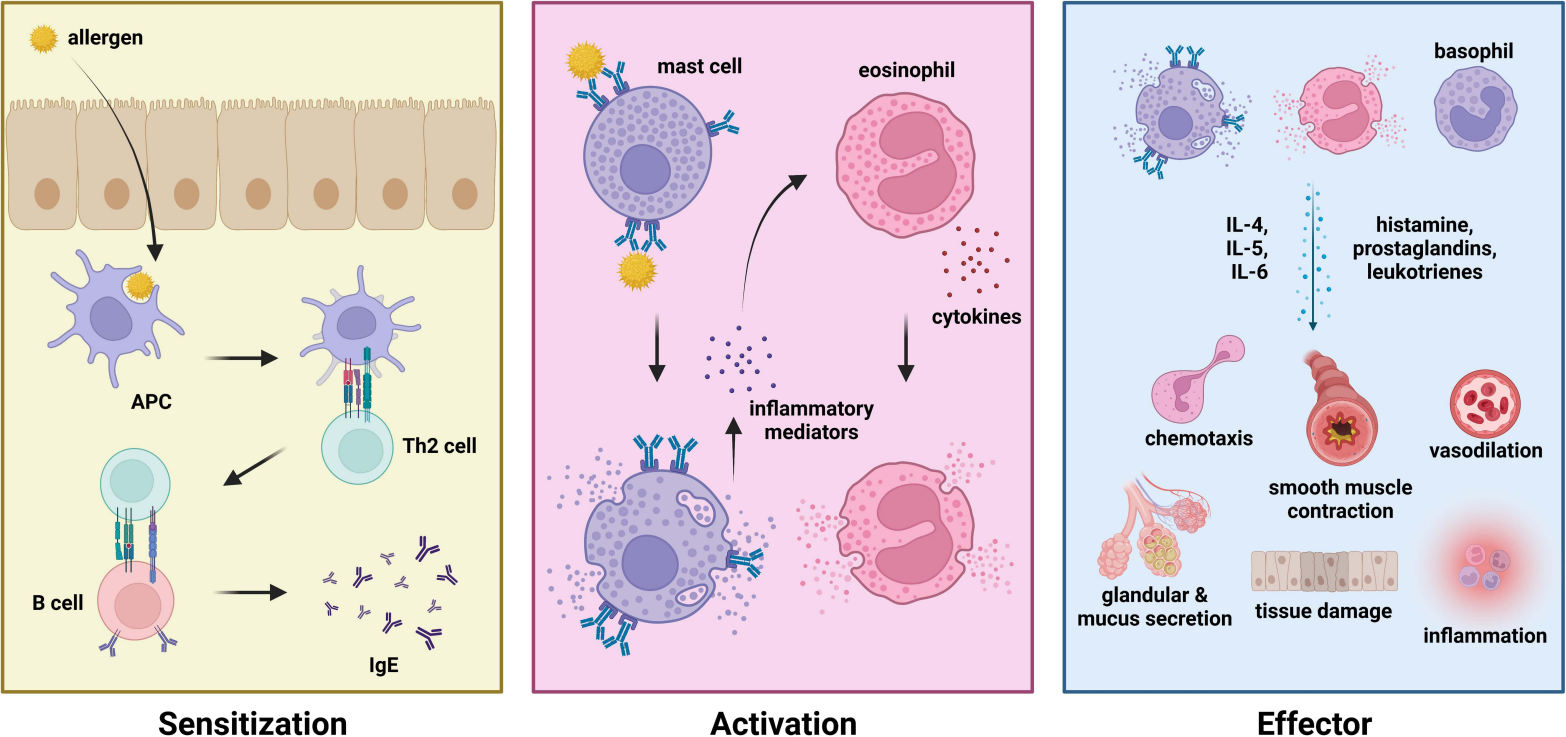
Phases of allergic disease—sensitization (**left**), activation (**middle**), and effector (**right**) phase.

Various components of the immune system have been identified to play essential roles in mediating allergic diseases. The IgE arm of humoral immunity is one of the most prominent drivers of allergic diseases such as allergic rhinitis, allergic asthma, and anaphylaxis. IgE-mediated allergic diseases are classified into three phases—namely the (1) sensitization phase, (2) activation phase, and (3) effector phase (Figure 1). During the sensitization phase, an allergen (i.e., pollen, dust mite dander, food) enters the body via the mucosal surfaces (i.e., respiratory tract, gastrointestinal tract). Upon penetrating the mucosal barrier, an antigen-presenting cell (APC) would take up the allergen, intracellularly degrade it into peptide fragments, and present the peptide onto MHC class II molecules. Phagocytosis of allergen particles are mediated by the presence of protein surfactants (i.e., surfactant protein A, surfactant protein D) which differ in distribution and variety across different types of allergens (33, 34). Upon presenting the allergen peptide to a CD4+ or helper T cell (i.e., Th2), these cells would gain their effector function to stimulate naïve B cells to differentiate into effector IgE plasma cells. Activated plasma cells would then secrete IgE which would subsequently bind to allergens upon re-exposure or challenge during the activation phase. Upon binding of IgE to allergen via the Fab region, effector cells with the corresponding FcεRI or FcεRII receptor would bind to the Fc region of bound IgE thereby activating the effector cell. These effector cells include the mast cells, eosinophils, and basophils. IgE cross-linking was also shown to drive allergic disease progression by impairing monocyte phagocytosis, leading to a pro-inflammatory microenvironment (35). These immune cells carry out various effector functions which are characteristic to the symptoms of different types of allergic diseases via the release of effector molecules (i.e., prostaglandins, leukotrienes, histamines) and the recruitment of other immune cells (i.e., dendritic cells) via cell signaling and chemotaxis to produce a state of inflammation (36, 37). These symptoms include upregulating inflammation, eosinophilia, smooth muscle contraction, excessive mucus secretion, vasodilation, and tissue damage (38). Moreover, various types of alveolar macrophages have also been identified as key regulators of respiratory allergic diseases (39).

One factor identified to affect the host immune system are sex differences due to hormonal variations. In general, findings suggest that there is increased prevalence of allergic disease across females than in males. One study from Ejima et al. demonstrated the alleviating effect of androgens on airway inflammation, highlighting the role of male sex steroid hormones (i.e., dihydrotestosterone) in suppressing type 2 cytokine production (40). A similar study by Fuseini et al. demonstrated the signal attenuation of house dust mite-induced type 2 and IL-17A inflammatory factors by the action of testosterone (41). Finally, the presence of sex-specific leptin and leptin receptor polymorphisms may also contribute to differences in asthma severity (42). Although the role of estrogen in allergic disease progression has been implicated, the results from such studies are inconclusive on whether the hormone leads to an ameliorative effect by inhibiting inflammasome activation or a pathologic effect by promoting type 2 proinflammatory cytokine production (43, 44).

Despite this, aberrations in the production of IgE leading to pathologically low levels of serum IgE has also been implicated to play a role in allergic disease progression. This condition is described as selective IgE deficiency, and a small number of studies have demonstrated the potential role of deficiency of serum IgE in potentiating various allergic diseases and worsened respiratory function (45). Apart from asthma, this syndrome may also lead to greater risk of other infectious (e.g., upper respiratory tract infection, pneumonia) and non-infectious disease (e.g., bronchiectasis, autoimmune disorders, arthritis), indicating the important role of maintaining a homeostatic level of IgE (46).

### 3.2. Th1/Th2 interplay

One of the primary mediators of allergic disease is a preferential shift towards Th2 predominant immunity, leading to unregulated inflammation and destructive immune responses. This has been described as the equilibrium model of immunity by Eberl, which highlights the involvement of a dynamic equilibrium between four mutually inhibitory branches of immunity. In the context of allergies, type 2 responses comprise immune cells and other mediators against large parasites (e.g., helminths) and fungi, which are also involved in allergic responses (47). Th1 immunity is typically characterized by beneficial immune responses, leading to better disease outcomes whereas Th2 immunity is associated with worsened disease outcomes, due to the uncontrolled production of proinflammatory cytokines, leading to destructive effects on the host. Although Th1 is known to antagonize Th2 responses, predominance of either Th1 or Th2 immunity was shown to lead to debilitating disease outcomes, such as acute lung pathology, airway hyperreactivity, and chronic inflammation (48). Therefore, it is necessary to devise ways to restore and promote Th1/Th2 balance across patients with allergic diseases.

To improve disease outcomes and control disease progression, several attempts at investigating strategies to regulate Th1/Th2 balance have been done in the context of allergic diseases. This involves the use of the purified carbohydrate, L-arabinose, as a treatment option among patients with wheat allergies, protein S among patients with bronchial asthma, and vitamins such as 1α,25-dihydroxyvitamin D_3_ against allergic rhinitis (49–51). Immunotherapy has also been identified as a viable strategy in controlling the production of proinflammatory cytokines associated with Th1/Th2 imbalance (52). A better understanding of this complex interplay between the two branches of helper T-cell mediated immunity can help in the development of more robust therapeutics.

## 4. Genetic factors of allergic diseases

### 4.1. Genetic predisposition

Several genes have been investigated for their involvement in allergic reactions. However, due to the heterogeneity of the response and how the disease tends to vary from person to person, an exact genetic basis has yet to be fully elucidated for allergic disease predisposition and severity. Several attempts have been made to characterize genes involved in inflammation and maintaining the integrity of the mucosa. Overall, the genes that were identified to be involved with allergic disease severity, progression, and development primarily function in (1) regulating inflammatory responses (i.e., IFN-α, TLR-1, IL-13, IL-4, IL-5, HLA-G, iNOS), (2) maintaining the vascular endothelium and mucosal lining (i.e., *FLG*, *PLAUR*, *CTNNA3*, *PDCH1*, *COL29A1*), (3) mediating immune cell function (i.e., *IL1RL1*, *PHF11*, *H1R*, *HDC*, *TSLP*, *STAT6*, *RERE*, *PPP2R3C*), and (4) affecting susceptibility to allergic sensitization (i.e., *ORMDLR3*, *CHI3L1*) (42, 53–58). Several studies have attempted to characterize the genetic profiles of individuals predisposed to and affected with allergic disease based on their polygenic architecture. Certain loci were identified to be associated with allergic disorders exclusively (e.g., *MIIP*, *CXCR4*, *SCML4*, *CYP1B1*, *ICOS*, *LINC00824*) whereas other pleotropic loci were shown to be associated with both autoimmune and allergic disorders (e.g., *PRDM2*, *G3BP1*, *HBS1l*, *POU2AF1*) (59). These genes shared a common genetic pathway being involved in inflammation found across different epithelial tissue types (i.e., skin, esophageal, vagina, lung) due to systemic circulation of allergic mediators, indicating the presence of shared genetic components that directly contribute to asthma and allergic disease pathogenesis (60).

Studies on the transcriptomic profiles of atopic individuals revealed the distinctive role of IL-13-associated disease pathways leading to eosinophilic airway inflammation and remodeling, resulting in persistent airflow limitation that is characteristic of allergic asthma. However, gene signatures varied significantly and could be compartmentalized based on function—with expression of genes involved in inflammation being limited to the superficial layer and lumen of the airway whereas genes involved in airway remodeling were limited to endobronchial biopsy samples (61). This distinct pattern of enriched gene signatures was also clearly observable across nasal brushing, sputum, and endobronchial brushing samples. These genes were primarily involved in eosinophilic airway inflammation, mast cell degranulation, and group 3 innate lymphoid cell function, resulting in adult-onset severe asthma (62). Further investigations of the nasal transcriptome revealed other genes involved in airway mucin production (i.e., *MUC5AC*) and mucus metaplastic transformation of airway epithelium (i.e., *FOXA3*) causing airway obstruction (63). This compartmentalization of enriched gene signatures presents a novel direction for the development of precision medicine-based therapeutics. However, there still exists numerous gaps in our understanding of the connections pathobiological mechanisms of allergic diseases. Further investigations must elucidate the interactive components involved in allergic diseases, thereby integrating the genetic, immunological, and pathophysiological aspects of allergy.

### 4.2. Epigenetic basis

The involvement of the epigenome in the pathophysiology of allergic diseases has recently been described and is attributed as one of the major linking factors of allergen and pollutant exposure to disease progression (64–67). Differential methylation of various loci due to exposure to cigarette smoke extract and allergen exposure leading to rapid lung decline, airflow limitation, and overall disease exacerbation (64). DNA methylation of specific genes involved in innate immunity (e.g., IL-1B, IL-6), Th1/Th2 balance (e.g., IL-4, IL-12B, IL-2) and other immune processes (e.g., BDNF, IL-17F, CXCL12, CCR7, RUNX1, CD3E, SERPINE1) have been identified in the progression of peanut allergies (65). Aberrations in DNA methylation have also been shown to be associated with airway epithelial cell dysfunction. Fluctuations in the DNA methylation signatures, due to varied exposure to epigenetic modulatory factors (e.g., traffic-related air pollution, allergen exposure), in genes involved in immune cell function (e.g., CD4+ T cells) and allergic disease pathogenesis (e.g., DUSP22, WTN7B) may also serve as the basis for the heterogeneity of symptoms of asthma and seasonal allergic rhinitis (68, 69). In the context of childhood allergic diseases, nasal DNA methylation signatures in three CpG sites were associated with changes in activated T cell and macrophage populations in the nasal mucosa, leading to allergic inflammation. These DNA methylation signals were also associated with IgE sensitization driving various allergic symptoms (70).

Similarly, the effect of histone acetylation has also been described. Using an IgE-mediated cow’s milk allergy mouse model, reduced percentages of Treg cells were associated with decreased levels of H3 and H4 histone acetylation across specific Treg cell loci. In the same study by Alhamwe et al., decreased histone acetylation was also observed across Th1 loci, preceding the typical reduction of Th1 cell populations associated with allergic responses. This epigenetic mechanism may be involved in a downregulatory mechanism that results in dysregulation of the type 1/type 2 immune response balance (71). On effector cells (e.g., mast cells), a study by Krajewski et al. describes alterations in histone acetylation that regulate the activation of mast cells involved in food allergies. Inhibition of the histone deacetylase with trichostatin A, a broad-spectrum histone deactylase inhibitor, led to changes in the intestinal cytokine profile, decreased expression of FcεRI and prevention of mast cell degranulation (72).

## 5. Macroecological factors associated with allergic disease

### 5.1. Exogenous early-life determinants

Several environmental factors have also been identified to lead to an increased risk for developing allergies. A study by Adami et al. confirms the involvement of biota depleting factors in worsening the severity of house dust mite-induced asthma, following administration of antibiotics. By intermittently allowing exposure to antibiotics during the early stages of life, this leads to several debilitating effects on the host—airway eosinophilia, airway hyperreactivity, and reduction in pulmonary Treg cell populations which would lead to a decrease in microbiome diversity and depletion of pro-regulatory species of microbiota (i.e., *Lachnospira* sp.) (73). Moreover, early life and prenatal exposure to different types of chemical compounds derived from polycarbonate plastics (e.g., bisphenol A) have also been shown to heighten the risk and severity of allergic diseases. Exposure to endocrine-disrupting chemicals has been shown to Th2/Treg cell imbalance, increased levels of IgE, and inflammatory cytokine production. Increased levels mRNA encoding of *GATA-3*, and decreased levels of mRNAs encoding *Foxp3* and *Helios* due to bisphenol A administration were identified as the primary mediators driving Th2 cell differentiation, leading to exacerbation of allergic asthma (74–76). Interestingly, the interaction of the hormone estrogen and exposure to bisphenol A has also been shown to contribute to increased risk of allergic disease associated with prenatal bisphenol A exposure (74). This pattern was also shown to be present in a study that investigated maternal exposure across different types of bisphenols (i.e., bisphenol F, bisphenol F). The findings of the study revealed a dose and sex-specific effect of bisphenol exposure leading to changes in the mucosal and systemic immune system, leading to impaired immunoregulation and developmental immunotoxicity (76). Overall, these studies implicate the importance of appropriate prenatal and antenatal practices in improving allergic disease outcomes.

### 5.2. Pollution

Worsening of air quality due to the accumulation of air pollutants and atmospheric particulate matter is one the most prominent factors driving worsened allergic disease outcomes among urban communities. The effect of pollution on allergy pathogenesis manifests in different ways—through the accumulation of reactive oxygen species leading to oxidative stress, enhancement of Th2 responses, upregulation of IgE production, eosinophilia, and impaired mucosal barrier function. Firstly, Jung et al. highlighted in their study the impact of environmental pollutants such as diesel exhaust particles, showing that exposure led to increased levels of the proinflammatory cytokine, IL-17, and worsened disease outcomes (77). Particulate matter and other air pollutants, which are known to carry microbes and viruses from the environment, found in haze are associated with house dust mite allergic sensitization. These atmospheric pollutants are involved in triggering and aggravating cellular inflammatory responses by stimulating sIgE production, contributing to the build-up of oxidative stress, and impairment of mucosal barrier function. (78). A study by Fernandes et al. showed that exposure to smoking and household air pollution also led to worsened disease outcomes across asthmatic adults. Many lifestyle practices such as domestic wood burning, which increased exposure to wood stove smoke, have debilitating effects on lung function, sensitivity to inhaled corticosteroids, and augments airway inflammation, leading to worsened disease outcomes. Across many urban communities, most individuals are subjected to dual exposure to household pollution and smoking, leading to harmful additive effects (79). Therefore, the control of biomass combustion, smoking prevalence, and the production of toxic exhaust fumes are necessary in order to reduce the harmful effects of pollutants.

### 5.3. Global environment

The dispersion of environmental allergens has also been implied to be responsible for variations in the global distribution of allergic disease. Aeroallergens (e.g., pollen, fungal spores) have increased in atmospheric abundance due to urbanization and green architectural practices. The increased public health risk for allergic diseases across developed countries may be attributed to two major driving factors—reduced biochemical diversity of pollen allergens and increased atmospheric pollen counts (80–82). These factors also shape the host microbiome composition, due to micro-ecologic related to lifestyle and the immediate surrounding environment. Seasonal changes in the incidence of allergic symptoms have also been reported, due to fluctuations in atmospheric pollen counts, domestic aeroallergens, and mite allergens across various geographic locations (83). Across temperate countries, there exists a distinct seasonality of different allergic diseases (e.g., allergic rhinitis, asthma, allergic conjunctivitis, atopic dermatitis) due to changes in daily temperature and humidity across different seasons (i.e., spring, summer, autumn, winter) (84). In contrast, among tropical and subtropical countries, a smaller subset of allergen classes typically predominates leading to an increased prevalence of a narrower spectrum of allergic diseases (85). Anthropogenic climate change, leading to increased global temperatures, has led to longer and more widespread pollen seasons, and increased pollen load—resulting in greater exacerbation and negative impacts on the respiratory health of allergic individuals (86, 87). Therefore, strategic design of residential and green spaces must consider the interactions of the type (e.g., temperate, subtropical, tropical) and quality of the ecosystem, its impact of air and soil pollution, and the impact on allergic disease incidence.

## 6. Microecological factors associated with allergic disease

### 6.1. Human microbiome

**Figure 2:**
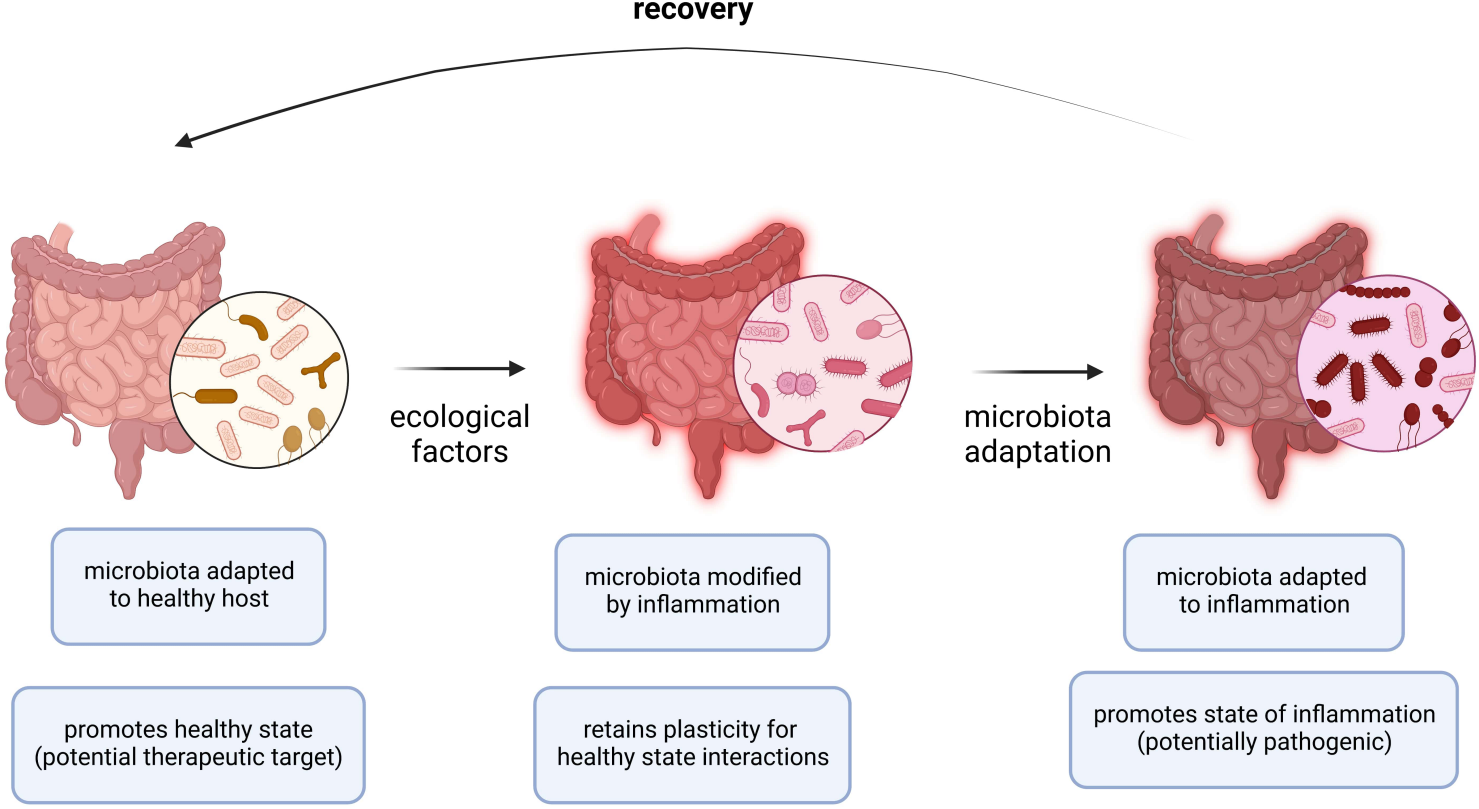
Adaptation of microbiota to allergic inflammation. During allergic inflammation, the microbiota undergoes changes as a result of a complex interplay between internal host and external environmental factors. In a healthy host or microbiota ecosystem, the microbiota is adapted to the healthy host and a healthy state persists. In periods of acute inflammation, ecological factors (i.e., allergen exposure, viral infection) cause the microbiota ecosystem to become inherently unstable and is modified by the immune effector responses while retaining a level of plasticity to preserve interactions occurring in the healthy state. In periods of chronic inflammation (i.e., allergies), the microbiota is adapted to inflammation and the state of inflammation persists. Recovery requires resuscitation from the chronic state of inflammation by restoring the normal endogenous communities comprising the host microbiota.

Despite its established significance in the context of other diseases, the microbiome has only recently gained attention in terms of its involvement in allergic disease outcomes. A stable microbiome has generally been shown to be associated with less severe forms of allergic diseases (88, 89). The biota alteration theory attributes change in endogenous microbial communities to inflammatory diseases, of which allergies have been described in detail. Several factors are involved in reshaping the plasticity for healthy state interactions, the stability of the microbial composition, and the adaptability to inflammatory responses (Figure 2). Moreover, there exists a standard microbiota composition that is typically altered in diversity and abundance of specific microbial species leading to pathologic states. The microbial species typically associated with healthy states include *Prevotella* sp., *Lactobacillus* spp., and *Bacteroides* spp. Therefore, the pathology of allergic diseases has been reshaped to recognize the microbiota as a key player in directing the host response and disease outcome (89).

Recent studies on the human microbiome have attempted to characterize the composition of the gut microbiota in association with the systemic manifestations of various allergic diseases. This would include diseases such as allergic eczema and hives, and more common diseases such as allergic rhinitis. According to Su et al., certain *Bacteroides* sp., together with *Romboustia* sp. and *Sutterella* sp. were associated with the disease eczema. In contrast, species correlated with allergic rhinitis consisted of Clostridia bacteria, Ruminococcaceae bacteria, Lachnospiraceae bacteria, *Eubacterium coprostanoligenes*, and *Atopobium* sp., all of which differed in abundance across allergic individuals (88). For skin allergies, *Staphylococcus aureus* has been shown to affect the severity and progression of skin allergies. Firstly, enterotoxins damage the epithelial lining of the skin, resulting in impairment of the protective barrier. As a result, a state of inflammation would occur and be maintained by the bacteria (90). Furthermore, the intake of specific dietary factors may also serve as adjuvants to allergic disease-associated immune responses, leading to sensitization against certain foodborne allergens. This, in turn, leads to the production of harmful bioactive metabolites that causes disruption of the gut barrier. Thus, the systemic manifestation of allergic diseases (i.e., food allergies) may be attributed to loss of structural integrity of the gut epithelial lining and increased gut permeability to inflammatory mediators (91). Other factors which have been identified to contribute to increased risk for allergic diseases due to changes in host microbiota include cultural factors such as a sedentary lifestyle, and nutritional factors such as deficiency in vitamin D (i.e., lack of exposure to sunlight), iron, and other essential micronutrients (25). Alterations in the host microbiota cause changes in levels of several bioactive metabolites that affect the host immune system, leading to changes in the progression of allergic diseases (92–95). Hence, Meyer et al. suggests vitamin and mineral supplementation to reduce the incidence and severity of food allergies among malnourished children (94). Additionally, Trompette et al. described the association of increased levels of circulating short-chain fatty acids (e.g., propionate) and protection against allergic inflammation in the lung (96). In effect, the importance of proper nutrition to attain a balance of microbiota-derived metabolites and improve overall gut microbiota metabolism is emphasized in allergic diseases. By the perspective posed in the biota alteration theory, the need to elucidate the interactions of the host microbiota and the role various metabolites play in allergic disease progression could help to augment the development of microbiome-targeted therapies.

Apart from the gut microbiome, several studies have attempted to characterize the differences in the microbial composition of other regions of the body, such as the respiratory microbiome. A study by Che et al. attempted to characterize and compare the nasal microbiome of individuals with allergic rhinitis to those who were unaffected. Findings from their study revealed that the presence of species such as *Vibrio vulnificus* and *Acinetobacter baumanni* in the nasal microbiome allergic individuals led to an increase in allergic mediators (e.g., GM-CSF, IFN*β*, IL-27, IL-1β), leading to allergic rhinitis and asthma (97). Differences in the microbial composition of individuals with respiratory allergies were evident in the nasopharyngeal microbiome, presenting a possible predictor for allergic disease progression in early childhood. Dysbiosis of the nasopharyngeal microbiome was shown to potentiate respiratory allergic responses, with the predominance of *Moraxella* sp. leading to induced pulmonary epithelial damage and increased proinflammatory cytokine expression (98). The microbiota composition of the upper and lower respiratory tract was also shown to be associated with allergic respiratory tract diseases, in a study by Cui et al. From the findings of their study, bacterial species such as *Haemophilus* sp., *Streptococcus* sp., *Staphylococcus* sp., and *Clostridium* sp. were shown to be associated with inflammation in the lower respiratory tract, parallel to their effect in the gut microbiome. Additionally, pharyngeal colonization of *Haemophilus* sp. and *Streptococcus* sp., together with the upper respiratory tract pathogen *Moraxella* sp., was identified as a risk factor for acute and aggravated allergic asthma episodes among children (99). Finally, the impact of synergistic infections with respiratory pathogens, such as the influenza A virus and *Streptococcus pneumoniae* was also implicated to interact with factors associated allergic inflammation to *Aspergillus fumigatus*. These findings indicate a “triple-disease” burden linking asthma, influenza virus infection, and pneumococcal pneumonia in the context of respiratory health (100). Although underlying immunologic responses and host cell signaling pathways may exist that may explain the complex host-pathogen-microbiome interactions present, these have yet to be fully elucidated. Overall, current studies reinforce the existence of a multifactorial crosstalk between different synergistic microbial factors, which may explain the variations in allergic disease progression.

### 6.2. Indoor microbiome

The effect of the distribution and abundance of microorganisms (e.g., bacteria, fungi) and their derivatives (e.g., endotoxins, spores) in the indoor microbiome has also been characterized in terms of the immunopathogenesis of asthma and exacerbation of airway inflammation through cytokines mediating type 2 immune cell responses (101–104). Several studies across various geographic regions have attempted to characterize the indoor microbiome across different populations, in relation to allergic disease severity. One study done in Malaysia identified various bacterial (i.e., *Sphingobium* sp., *Rhodomicrobium* sp., *Shimwellia* sp., *Solirubrobacter* sp., *Pleurocapsa* sp.) and fungal species (e.g., *Torulaspora* sp., family Leptosphaeriaceae) with protective roles in the context of respiratory health and asthma severity across tropical areas. Similarly, several species of bacteria (e.g., *Izhakiella* sp., *Robinsoniella* sp.) were also identified to be associated with asthma exacerbation. Factors which were identified to shape the indoor microbial composition include building infrastructure, the abundance of common household insect pests, and the presence of molds (105). Two separate studies in different schools across China investigated the association of the indoor microbiome and the severity of various allergic diseases (e.g., rhinoconjunctivitis, asthma, rhinitis, eczema). Bacteria such as *Prevotella* sp., *Lactobacillus iners*, and *Dolosigranulum* sp. were shown to induce a protective effect against rhinitis for preschool children. In contrast, fungi such as *Aspergillus subversicolor* and bacteria such as *Collinsella* sp. and *Cutibacterium* sp. were associated with worsened disease outcomes for asthma, rhinitis, and eczema respectively. These opportunistic pathogenic species are often associated with chronic inflammatory diseases and unregulated immunoactivation due to the production of highly potent virulence factors (e.g., cAMPs, porphyrins, hyaluronate lyase) across different mucosal surfaces of the body. Differences in the microbial composition of urban and rural schools were also shown to be a primary factor in shaping the prevalence of various allergic diseases. For example, species of *Brachybacterium* was more common across rural areas and was generally associated with improved disease outcomes across high school children with allergic rhinitis. In contrast, potentially pathogenic species of microbes such as *Pseudoalteromonas* sp*.*, *Microbacterium foliorum*, a prominent member of the phyllosphere microbiome, and the protist, *Neospora caninum*, were associated with increased incidence of wheeze, rhinitis, and rhinoconjunctivitis. These microbes were shown to interact with different components of the immune system, leading to Th1/Th2 imbalance and IgE antibody production (106, 107). Findings from a study in the United States revealed that other factors involved in altering fungal allergen load (i.e., *Aspergillus* sp., *Alternaria* sp.) include the alpha diversity of bacterial species (e.g., *Staphylococcus* sp., *Porphyromonas* sp., *Moraxella* sp., *Sutterella* sp., *Clostridim* sp., family Neisseraceae), window opening, and the presence of pets and flowering plants within the vicinity. From these studies, it was established that the microbial species which are most prevalent in an area are referred to as the “core microbiome” (108). The exact influence of changes in the core microbiome and an individual’s susceptibility to allergic disease due to allergen load and changes in host metabolic and immunologic profiles remains to be fully elucidated.

### 6.3. Virus respiratory infections

Several types of viral infections have been shown to worsen and exacerbate allergic disease outcomes. This is particularly relevant in cases of respiratory allergies, with rhinoviruses, respiratory syncytial viruses, influenza viruses, and coronaviruses identified as exacerbating agents of allergic diseases. For one, adult asthma exacerbations were associated with viral respiratory infections (VRIs) caused by human rhinovirus, human metapneumovirus, influenza virus, and respiratory syncytial virus (109–115). Other factors involved include seasonal variations in viral infection incidence and the genetic divergence of viruses across populations. As a result, the incidence of VRIs were identified as a predictive factor for adult asthma exacerbations in patients across various seasons (116). Conversely, atopic individuals were shown to be predisposed to more severe viral respiratory diseases. The mechanistic basis for the interactions of VRIs and allergic disease progression may be attributed to several factors that are still being investigated. Current findings, however, suggest that respiratory viruses prevent the development of immune tolerance and enhance allergic sensitization to aeroallergens. This results in increased inflammation and hyperresponsiveness in the respiratory tract. As a result, the airway mucosa becomes significantly more permeable to penetration by allergens, leading to various effector functions characteristic of respiratory allergic diseases (117, 118). Additionally, certain viruses (i.e., rhinoviruses) have also been implicated in persistent Th1/Th2 imbalance among patients with allergic asthma (119). Therefore, understanding the complexity surrounding the relationship of VRIs and allergic diseases must be integrated with the growing incidence of viral pathogens (i.e., SARS-CoV-2).

## 7. Therapeutic and control strategies for allergic diseases

Within the past decade, several treatment methods have been investigated for their applicability in clinical practice, ranging from the use of pharmaceutical drugs, inhalers, nebulizers, and other immunomodulatory and immunotherapeutic agents (120, 121). Some studies also describe a competition between IgG and IgE antibodies for antigenic sites on specific allergens, with IgG binding leading to desensitization of the host to the specific allergen due to reduced binding sites for IgE. However, the exact mechanistic basis for the competition of IgG and IgE binding and how this can be exploited further to develop new prevention strategies remains poorly elucidated (122, 123). Current findings, however, indicate the involvement of low affinity IgG and its interactions with the inhibitory receptor, Fc*γ*RIIb, leading to inhibition of mast cell degranulation (124).

Various public health programs against allergies have been established and implemented globally. While allergen avoidance initially persisted as the primary prevention strategy for allergic diseases, recent findings have suggested the opposite—repeated exposure to allergen can lead to attenuation and possibly eradication of severe disease symptoms. To address the ecological aspect of allergic disease incidence, urban ecosystems must be designed to maximize biodiversity of native, non-allergenic vegetation and grassland species (81). Seasonal variations due to geographic differences must also be considered in the implementation therapeutic strategies (125).

### 7.1. Pharmaceuticals and biologics

Most pharmaceutical drugs typically target a component involved in mediating the allergic response by disrupting or abrogating its function or inducing a tolerogenic state within the host. For example, leukotriene receptor antagonists which target and suppress cysteinyl leukotrienes that function in eosinophilia, inflammation, and airway hyperresponsiveness associated with viral-induced asthma exacerbation have been identified as effective treatments (126). Antihistamines are another prominent classification of anti-allergy drugs routinely used in clinical practice. This class of pharmaceuticals mainly act by antagonizing the action of smooth muscle cells by stimulating histamine action in the H1-receptors (127). However, a prominent adverse reaction often associated with the use of antihistamines is sedation, which is mainly found in first-generation antihistamines. This is due to low brain uptake when bound to blood proteins such as serum albumin (128). Second-generation antihistamines (e.g., bilastine, loratadine, desloratadine, cetyrisine, levocetirisin) remain as one of the most widely available pharmaceutical drugs for allergic diseases worldwide (129). Many attempts have been made to further characterize the interactions of such widespread pharmaceutical drugs in the context of different types of allergic diseases. A pharmacokinetic study on cetirizine, one of the most widely distributed and cost-effective antihistamine, revealed zwitterionic and lipophilic properties which possible serves as the basis for the differences in antihistamine potency (128). Pharmacogenomic studies revealed the association of certain gene polymorphisms (e.g., CRTH2) and the effective dosage of antihistamine needed to alleviate symptoms of allergic diseases (e.g., chronic urticaria) (130). Investigations on the different molecular properties of the molecule can enable further improvement of the efficacy and potency of pharmaceuticals as treatments for allergic diseases.

In terms of biologics, the humanized recombinant IgG monoclonal antibody, omalizumab (i.e., XOLAIR), has emerged as a prospective therapeutic agent to effectively treat and mitigate IgE-mediated allergic diseases. It functions by targeting IgE to prevent the activation of effector cells which cause the symptoms of allergic disease (131, 132). This causes a competitive binding inhibitory effect, leading to IgE clearance and immune cell inactivation. This is carried out through an exploitation of the intrinsic flexibility of IgE, leading to accelerated dissociation. The presence of specific structural features in IgE, when compared to other antibody isotypes, such as the C*ε*2 domains and globule-like properties of the Cε3, enable the formation of an allosteric communication pathway to prevent the simultaneous binding of IgE to both of its receptors (i.e., FcεRI, FcεRII). This property would, therefore, prevent allergen-independent activation of mast cells due to crosslinking of FcεRI-bound IgE by CD23. However, the cost of production and resources required limits the widespread distribution and effective implementation of this biologic drug as a treatment for allergic diseases (133). Beyond logistical and economic constraints, there still exists a lack of understanding on the basis for dissociation of allergen specific IgE on mast cells caused by the anti-IgE molecule. Attempts to further circumvent the inherent limitations of omalizumab treatment was done through the development of an omalizumab biobetter antibody with improved stability, binding affinity, and efficacy when compared to the standard omalizumab molecule (134). Despite the pathological role of IgE being implicated by the function of IgE, adverse reactions to omalizumab have also been reported due to the formation of immune complexes between the monoclonal antibody and its target IgE antibody, leading to the manifestation of skin inflammation and anaphylaxis through an IgG receptor-dependent mechanism (i.e., Fcγ) (135).

Ligelizumab, another type of monoclonal anti-IgE antibody, is also being prospected for its enhanced neutralization of free serum IgE, inhibition of IgE binding to FcεRI, basophil activation, IgE production by plasma cells, and prevention of passive systemic anaphylaxis when compared to omalizumab. However, omalizumab remains more potent in terms of inhibiting IgE:CD23 interaction to prevent allergen presentation and other transport processes associated with allergic responses (136). Beyond omalizumab and ligelizumab, several other attempts at developing improved anti-IgE biologics (e.g., UB-221, 8D6, MeDI4212) for treatment of various allergic diseases have also been made but have yet to reach late-stage clinical trials (137–139).

Apart from anti-IgE biologics, Schanin et al. report the use sialic acid-binding immunoglobulin-like lectin immunoregulatory receptor (Siglec)-6 on mast cells as a potential target for therapeutic use. In their study, Siglec-6 monoclonal antibody clones were developed, with the AK04 variant being capable of inducing receptor cluster formation containing inhibitory phosphatase. The epitope-specific agnostic activity of the Siglec-6 mab prevented systemic anaphylaxis with a single dose and reduced overall mast cell activity with chronic dosing (140). Although the therapeutic strategy has shown potential in driving this occurrence, the lack of a mechanistic basis for its mechanism eludes its prospective use in clinical practice.

### 7.2. Microbiota-targeted therapies

**Figure 3:**
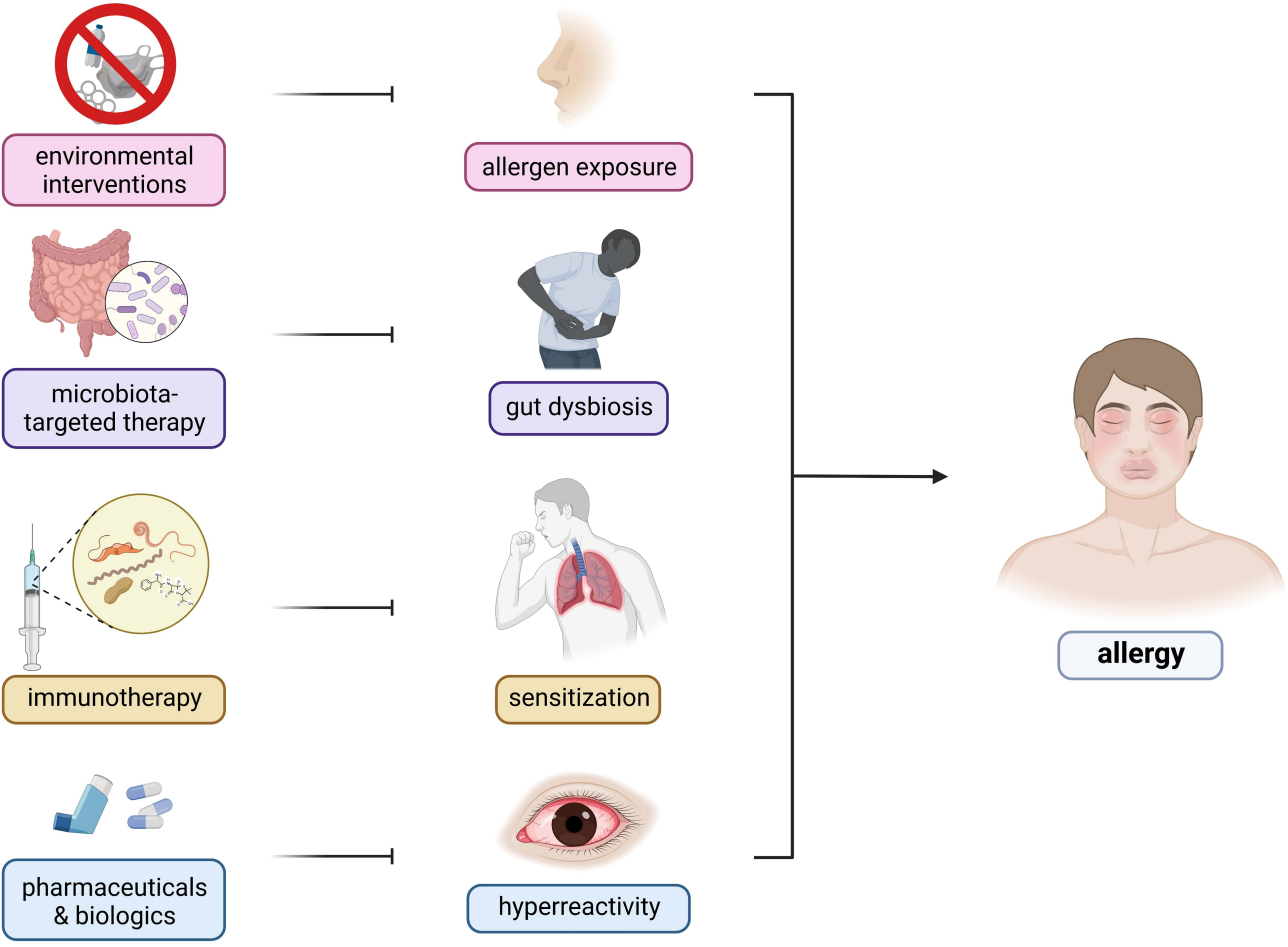
Therapeutic targets and strategies for allergic diseases. There are several therapeutic strategies being developed for the control and mitigation of allergic diseases—(1) environmental interventions, (2) microbiota-targeted therapy, (3) immunotherapy, and (4) pharmaceuticals and biologics. Environmental interactions include public health programs involving infrastructure development and changes to lifestyle factors. Microbiota-based therapies consist of prebiotic, probiotic, and synbiotic use to restore the host microbiome to reduce predisposition to inflammation. Immunotherapies involve repeated exposure to gradually increasing dosages of allergens to ameliorate the host immune response. Pharmaceuticals include traditional oral antihistamines, inhalers, and nebulizers to target specific components of the host immune response whereas biologics include monoclonal antibodies targeting IgE and other components of the allergic response.

Interestingly, the recognition of the involvement of the microbiome has enabled the investigation of the use of prebiotics and probiotics as attenuating agents against the progression of allergic diseases. However, further investigation is required to elucidate the exact causal mechanism and strategies for improving the efficacy of microbiome-based therapeutic for treatment of allergic diseases (Figure 3) (141). Current findings suggest that allergy attenuation can be achieved by microbiota-dependent upregulation of immunomodulatory Treg cell populations. Turner et al. describes the use of species of Clostridiales to induce the expression of TGF-*β*1 receptors among Treg cells, leading to regulate allergic disease progression (21). Similarly, Karimi et al. illustrated the potent immunoregulatory capacity of oral treatment with live *Lactobacillus reuteri*, leading to the attenuation of airway hyperresponsiveness and inflammation (142). Future studies that investigate the immunomodulatory potential of *Lactobacillus* sp. probiotics must consider the interactions these bacteria have on the amelioration of disease symptoms. The presence of immunosuppressive motifs across the genome sequences of probiotic species of *Lactobacillus* associated with reduced allergenicity have been identified and could serve as the basis for the selection of probiotic species to be utilized in treatment strategies (143). Additionally, previous investigations suggest prebiotic and probiotic supplementation to promote greater abundance of *Prevotella* spp. and *Bifidobacterium* spp., respectively, as microbiota-directed treatment strategies for atopic patients (144, 145). A study by Zhen et al. attempted to elucidate this relationship by correlating the presence of various bacterial enterotypes to the tryptophan metabolic pathways. Findings from the study revealed that the production of indole derivatives was associated with the attenuation of the disease. Indole-3-lactic acid and indole-3-butyric acid inhibited allergic pathogenesis by suppressing the production of IL-4 and IL-5 in macrophages. Indole acetic acid, on the other hand, was shown to be involved in balancing Th17/Treg responses, together with ILA. Plasma components, such as lysozyme C, cystatin-3, and kininogen-1 were also shown to decrease allergic symptom severity (146). Moreover, the consumption of dietary fibers as a prebiotic source may attenuate allergic disease symptoms by the production of short-chain fatty acids which would inhibit the formation of type 2 dendritic cells that would mediate allergic airway inflammation (96).

### 7.3. Immunotherapy

The emergence of immunotherapies as effective treatment strategies to various types of allergic diseases can be attributed to several factors impacting several aspects of the immune system. In a study by Scadding et al., patients treated with immunotherapies experienced less severe symptoms, had better respiratory function, and reduced levels of nasal fluid concentrations of IL-4, IL-9 and eotaxin, a potent eosinophil chemoattractant, after challenge with grass pollen (147). Immunotherapies have been tested against milk allergies via oral administration, for hymenopteran venom allergies, grass pollen allergies, and even house dust mite allergies (147–149). Jung et al. demonstrates in their study the viability of two different types of sublingual immunotherapy as a treatment strategy for patients with dust mite-induced allergic rhinitis, each with a distinct set of benefits (150). Similar findings were also reported by Matsumoto et al., wherein long-term immunotherapy with house dust extracts led to amelioration pulmonary function across asthmatic patients (148). Thus, the growing success of such studies highlights the potential of immunotherapies as viable preventive measures for severe allergic disease symptoms. However, safety concerns remain a primary roadblock to the effective implementation of immunotherapies in clinical practices. Additionally, adjuvant therapies using chitin, a major component of various allergy-causing organisms (e.g., house dust mites, crustaceans, fungi), may be utilized to improve the efficacy of therapeutics that attenuate the Th2 response (101). Current studies have attempted to compare different components of immunotherapy extracts that would produce the most desirable clinical outcomes. For example, a study by Du et al. attempted to compare the potency of house dust extracts from house dust mite allergen extracts. Findings from their study revealed greater allergenic potential of house dust mite allergen extracts, implicating its overall preferability over standard house dust extracts (151). The ability to exactly determine at what point and at what dosage re-exposure to an allergen would induce a beneficial, long-lasting, protective effect rather than a life-threatening, anaphylactic shock remains a major gap in current research and necessitates further investigation. The standardization of allergen immunotherapy extracts may improve the therapeutic efficacy birch, ragweed, dog hair, and *Alternaria* allergies, as highlighted by Due et al. (152).

Other prospective targeted therapies are also being explored for their capacity as immunotherapeutic strategies for allergic diseases. In the context of cow’s milk allergy, oral immunotherapy alleviated allergic symptoms and reduced levels of cow’s milk-specific IgE. However, adverse reactions were observed in several individuals on oral immunotherapy (153). As a potential alternative, the holoprotein form of the lipocalin beta-lactoglobulin (holo-BLG) protein is being investigated for its immunotherapeutic potential. Despite being a component of cow’s milk, which has been shown to induce allergies as well, this protein was identified as a novel component in inducing a farm protective effect against pollen allergies among farmers by promoting regulatory cell function and downregulating antigen presentation to effector cells. Prophylactic treatment with the protein results in disruption of specific IgE production and attenuated the type 2 response (154). Although not readily used or available for the clinical treatment of cow’s milk allergy, these findings on holo-BLG represent a myriad of potential directions for immunotherapeutic development for allergic disease mitigation.

An interesting prospect that could be undertaken in future research on immunotherapy potency is the combination of immunotherapeutic extract use with administration of biologics and other readily available treatments. A study by Bożek and colleagues showed that a combination of an allergen vaccine with the biologic, omalizumab, significantly increased the efficacy of allergen immunotherapy by reducing the incidence of asthma exacerbation and the daily dose of inhaled corticosteroids required by patients with house dust mite-driven asthma (155). Together, these findings highlight the importance of an integrated approach to provide complete amelioration of quality-of-life and improve overall disease outcomes for allergic individuals.

## 8. Discussion

The past century has been a revolutionary period for immunology, with new findings reshaping our understanding of the fundamental aspects of the immune system. From the discovery of underlying molecular pathways and mechanisms to the development of novel theories on the evolution of the immune system, these breakthroughs culminate in the current state of our global health scheme. This is especially relevant in the context of allergic diseases, which are regarded as one of the leading chronic inflammatory diseases globally.

**Figure 4:**
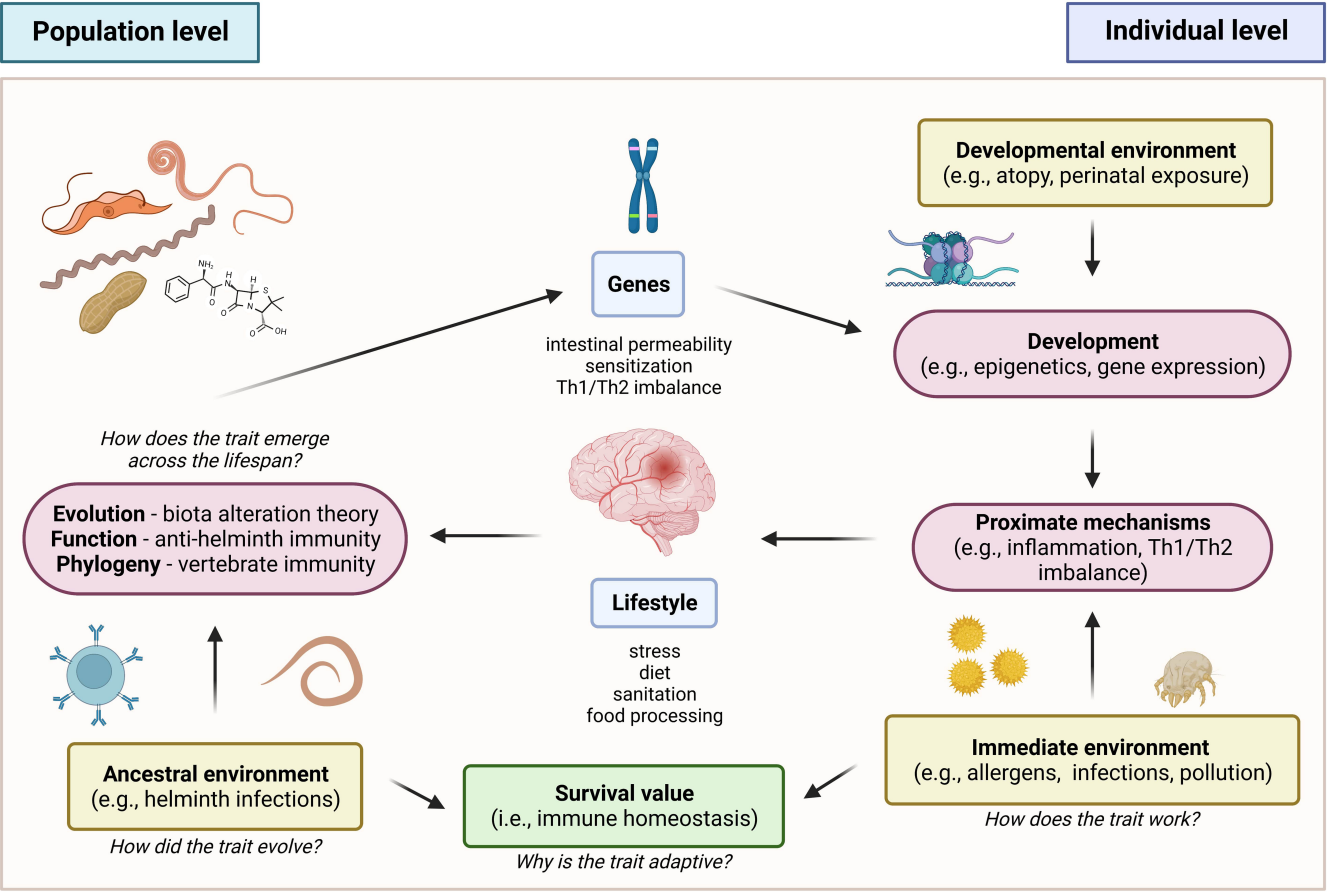
Proposed framework for allergic disease based on tinbergen’s four questions (156). In the context of evolutionary history, the ancestral allergic responses are postulated to be a form of antiparasitic immunity that persisted across vertebrates. In terms of development, the emergence of global de-worming efforts and improved sanitation practices resulted in changes in the persistence and primary function of the trait, masking its original function against helminthic parasites. This resulted in a mechanism involving misdirected responses against innocuous allergens. However, the trait persisted due to its conferred survival value for immune homeostasis.

To better understand the interrelationships of the various factors that underlie the pathogenesis and progression of allergic diseases, this review has adopted an integrative approach to correlate the historical, immunologic, and genetic aspects of the disease. Accordingly, a framework for these factors which utilized Tinbergen’s four questions is proposed (Figure 4) (156). In terms of evolutionary history, the ancestral allergic response is postulated to primarily be a form of antiparasitic immunity utilized by mammalian species, mediated primarily by the IgE immune responses. A primitive form of antiparasitic immunity is also exhibited in other vertebrates such as avian and non-avian reptiles mediated by the IgY antibody (4–6). However, the survival value for IgE responses has been largely masked with the advent of global deworming efforts, improved sanitation, and changes in global environmental conditions (16, 25, 157). Several theories postulating the basis of the continued persistence of the IgE responses, leading into allergic diseases, have been made in relation to the interactions of the immune system with host genetic, micro-ecologic, and macro-ecologic factors (18, 25). However, the concurrent evolution of these responses resulted in the reliance on a state of complete immunologic, genetic, and ecologic homeostasis as evidenced by the emergence of selective IgE deficiency syndromes that occur as a result of dysregulated immune function (45, 46). These observations highlight the importance of attenuating excessive, uncontrolled immune reactions and effector functions by restoring a proper balance (e.g., Th1/Th2, microbiome composition) in various aspects involved in allergic diseases. Therapeutics and control strategies at both the individual and macroscale level must consider this complex interplay of immunologic, genetic, and ecologic factors in order to better achieve desirable health outcomes without severely affecting other aspects.

In the pursuit of more effective diagnostic, preventive, and therapeutic strategies, a better understanding of the interplay of these factors is essential. Reshaping current perspectives about allergic diseases would help in the development of effective approaches in the global health scheme, to remedy the public health burden posed by such chronic conditions.
